# Editorial: Horizons in Systems Neuroscience 2022

**DOI:** 10.3389/fnsys.2024.1415569

**Published:** 2024-05-09

**Authors:** Wen-Jun Gao, Olivia Gosseries, Preston Garraghty, Mathew Diamond, Maria V. Sanchez-Vives

**Affiliations:** ^1^Department of Neurobiology, Drexel University College of Medicine, Philadelphia, PA, United States; ^2^Coma Science Group, GIGA-Consciousness, GIGA Institute, University of Liège, Liège, Belgium; ^3^Department of Psychological and Brain Sciences, Indiana University College of Arts and Sciences, Bloomington, IN, United States; ^4^Cognitive Neuroscience, Sector International School for Advanced Studies (SISSA), Trieste, Italy; ^5^Department of Psychological and Brain Sciences, Institut d'Investigacions Biomèdiques August Pi i Sunyer (IDIBAPS), Barcelona, Spain; ^6^Institucio Catalana De Recerca I Estudis Avançats (ICREA), Barcelona, Spain

**Keywords:** Systems Neuroscience, brain, theory, hypothesis, innovation

With its intricate network of billions of neurons, the human brain functions like a conductor, orchestrating thoughts, emotions, and actions. Systems Neuroscience aims to understand this complex symphony. Driven by technological advancement and a surge in interdisciplinary collaborations, this field has undergone a remarkable transformation.

The Research Topic “*Horizons in Systems Neuroscience 2022*” showcases fascinating new avenues of exploration. The nine articles cover diverse topics, including neural oscillations, perception, anxiety, memory, neuroplasticity, hypothesis, and theory. These works collectively provide insights into the brain's complexity and pave the way for future discoveries. All contributing authors were nominated in recognition of their prominence and influence in their respective fields.

Perception and actions are fundamental processes that characterize our lives and our possibility to modify the world around us. Bosco et al. reviewed the literature on how “*The influence of action on perception spans different effectors*.” The manuscript focuses on the influence of action on perception, specifically on the action planning and the phase following the execution of the action. The authors conclude that action planning and action execution constantly influence perception, which may be used to improve artificial intelligence (AI) systems and increase users' trust in AI.

At the systems level, recent research into neural oscillations spans different brain areas, species, and disciplines, granting us a common ground for the disparate fields of neuroscience. In a minireview, Miles et al. reviewed articles related to “*Hippocampal beta rhythms as a bridge between sensory learning and memory-guided decision-making*.” The authors highlight the role of beta oscillations in mediating coupling between the hippocampus and other regions involved in coordinating memory-guided behavior. This review puts forward hypothesis extending the role of beta oscillations beyond sensory systems toward a general role of hippocampal beta in enabling inter-regional coupling for sensory-driven, cue-reward associations and for enabling memory-guided behavior.

Long-term memory is achieved through a consolidation process where structural and molecular changes integrate information into a stable memory. In a review, Osorio-Gomez et al. discuss “*Transforming experiences: Neurobiology of memory updating/editing*.” It explains how long-term memory is formed and updated through a consolidation process involving structural and molecular changes. The process is dynamic, adapting to environmental changes and integrating new experiences. The article highlights the potential clinical implications of memory updating in conditions like drug addiction, phobias, and post-traumatic stress disorder.

Heck et al. further summarize the “*Cerebellar control of thalamocortical circuits for cognitive function: A review of pathways and a proposed mechanism*” in a minireview. The article explores the cerebrocerebellar interactions and cerebellothalamic pathways in cognitive and motor functions. It discusses the role of the thalamus in coordinating neuronal oscillations, indicating increased functional connectivity. The authors suggest that cerebellothalamic pathways may be crucial in coordinating neuronal communication.

In a systematic review, Mowery and Garranghty discuss “*Adult neuroplasticity employs developmental mechanisms*.” It summarizes studies showing adult neural plasticity, including primate somatosensory cortex. The article also discusses experiments revealing the physiological, morphological, and neurochemical mechanisms permitting this plasticity. It concludes that adult cells return to critical period-like plastic states under prolonged sensory deprivation.

Historically, spinal cord processes were considered mere mechanical relays for signals. Recent research challenges this view, this review by Grau et al. reveals that spinal cord mechanisms can organize behavior, alter pain processing, and infer stimulus relations. These processes resemble brain-dependent learning pathways. Spinal cord injury can induce plasticity while GABA transmission has a crucial role regulating such plasticity. Understanding spinal cord functions informs brain models and offers new treatments for spinal cord injury.

Anxiety disorders are the most common class of mental illness. A wealth of data has implicated the medial prefrontal cortex in the regulation of anxiety, and norepinephrine is a crucial neuromodulator of arousal and vigilance believed to be responsible for many of the symptoms of anxiety disorders. Bouras et al. reviewed “*Prefrontal modulation of anxiety through a lens of noradrenergic signaling*.” This article details the various potential projections and mechanisms through which the medial prefrontal cortex can exert executive control over subcortical regions involved in anxiety following locus coeruleus activation with the proposed model and fascinating future directions.

In a clinically relevant review, Bonin et al. summarize the “*Assessment and management of pain/nociception in patients with disorders of consciousness or locked-in syndrome*.” The authors discuss the challenges in assessing and managing pain in such patients. It highlights the need for clear guidelines and explores various topics, including the neurophysiology of pain, its impact, and treatment strategies. The review also suggests potential research directions for improving patient management.

Finally, as stated in the thought-provoking search for a theory summed up by Roland, the debate about “*How far neuroscientists are from understanding brains*” remains. This article highlights the current gaps in neuroscience, particularly in understanding how neurons interact at all scales and how brains function. It points out conceptual obstacles, such as the lack of models explaining neuron interactions, ambiguity in distinguishing different types of brain activities, and the insufficiency of dynamical systems theory to account for central nervous system activities. The author suggests that spatial dynamics could be a solution. The author also emphasizes the need for single-trial designs and statistics, as pooling and averaging data can destroy their underlying dynamics. The hypothesis/theory presented in the article is significant but perhaps also provocative, including the critical challenges identified and potential solutions proposed by the author. Nevertheless, it paves the way for the need for a theory explaining how the brains work.

These articles highlight the challenges in integrating findings across scales, deciphering the brain's code, and understanding its embodiment in the world. Despite these challenges, the diverse perspectives showcased in his Research Topic demonstrate the potential for groundbreaking discoveries in Systems Neuroscience. We eagerly await pioneering research that will sharpen the future of Systems Neuroscience.

Overall, the research and perspectives presented in this Research Topic underscore the complexity and interconnectedness of the brain's systems. By pushing the boundaries of knowledge, Systems Neuroscience has the potential to revolutionize our understanding of the brain and open new avenues for treating neurological and mental disorders.

## Author contributions

W-JG: Writing – original draft, Writing – review & editing. OG: Writing – review & editing. PG: Writing – review & editing. MD: Writing – review & editing. MS-V: Writing – review & editing.

